# Microvascular Dysfunction in Patients with Prediabetes: Novel Methods Identify Impaired Microcirculation

**DOI:** 10.3390/life16020326

**Published:** 2026-02-13

**Authors:** Stamatina Lamprou, Nikolaos Evangelidis, Nikolaos Koletsos, Ioanna Zografou, Anastasia Stoimeni, Gesthimani Mintziori, Vasileios Gkolias, Christina-Maria Trakatelli, Christos Savopoulos, Michael Doumas, Areti Triantafyllou

**Affiliations:** 1 First Propedeutic Department of Internal Medicine, Hypertension Outpatient Clinic and ESH Excellence Centre of First Propedeutic Department of Internal Medicine, AHEPA University Hospital, Aristotle University of Thessaloniki, 54636 Thessaloniki, Greece; stamatin@auth.gr (S.L.); evangeln@auth.gr (N.E.); csavvopo@auth.gr (C.S.); 2 Third Department of Internal Medicine, Papageorgiou General Hospital, Aristotle University of Thessaloniki, 54629 Thessaloniki, Greece; nick.koletsos@gmail.com (N.K.); ctrak@auth.gr (C.-M.T.); 3 Second Propedeutic Department of Internal Medicine, Hippokration General Hospital, Aristotle University of Thessaloniki, 54642 Thessaloniki, Greece; ioannazo@yahoo.gr (I.Z.); doumasm@auth.gr (M.D.); 4 Fourth Department of Pediatrics, Aristotle University of Thessaloniki, Faculty of Health Sciences, School of Medicine, Papageorgiou General Hospital, 54629 Thessaloniki, Greece; anastoim@gmail.com; 5 Unit of Reproductive Endocrinology, First Department of Obstetrics and Gynecology, Papageorgiou General Hospital, Aristotle University of Thessaloniki, 54629 Thessaloniki, Greece; gefsi@auth.gr; 6 Laboratory of Primary Health Care, General Practice and Health Services Research, School of Medicine, Aristotle University of Thessaloniki, 54124 Thessaloniki, Greece; vgkolia@auth.gr

**Keywords:** dysglycemia, early microvascular dysfunction, myocardial microcirculation, laser speckle contrast analysis, prediabetes, subclinical cardiovascular disease, subendocardial viability ratio, skin microcirculation

## Abstract

Background: Skin and myocardial microvascular dysfunction in prediabetes remains underexplored, and limited studies have investigated the microcirculation in prediabetes in multiple vascular beds. This study aimed to examine microvascular alterations in patients with prediabetes, patients with type 2 diabetes mellitus (DM), and normoglycemic controls without established cardiovascular disease (CVD). Methods: In this cross-sectional study, the microcirculation was assessed using established and novel noninvasive techniques. The skin microvascular reactivity was evaluated using laser speckle contrast analysis (LASCA). The myocardial perfusion was assessed by the subendocardial viability ratio (SEVR). The retinal microvasculature was evaluated using digital nonmydriatic fundus photography, the renal microvascular damage through the urinary albumin-to-creatinine ratio (ACR), and the peripheral vasculopathy by the augmentation index (AIx). Results: Sixty-seven participants were included (22 controls, 24 with prediabetes, 21 with DM; aged: 55.9 ± 9.4 years). Patients with prediabetes and DM showed significantly reduced baseline-to-peak skin flux responses in LASCA compared with controls (*p* = 0.006), and lower SEVR values (*p* = 0.001). Moreover, no significant differences were identified in the retinal, renal, or peripheral microvascular indices. In multivariate analysis, systolic blood pressure and glucose were independently associated with skin microvascular dysfunction, while the heart rate and arteriovenous ratio were associated with the SEVR. Conclusions: In this cross-sectional study, impaired skin and myocardial microvascular function were observed in patients with prediabetes in the absence of overt CVD. These findings suggest that LASCA and the SEVR may serve as sensitive markers for the detection of early, subclinical microvascular dysfunction in prediabetes.

## 1. Introduction

Diabetes mellitus (DM) is associated with an increased risk of death caused by cardiovascular disease (CVD) and a higher risk of all-cause mortality [1,2]. Morbidity and mortality in patients with DM are related primarily to micro- and macrovascular complications of the disease, along with the high prevalence of other traditional CVD risk factors, such as obesity, hypertension, and dyslipidemia [3]. Moreover, the prevalence of prediabetes is increasing worldwide [4]. Prediabetes has also been associated with an increased risk of CVD and all-cause mortality [5]. In the general population, prediabetes is associated with an increased risk of all-cause mortality, coronary heart disease (CHD), and stroke [5]. Subclinical microvascular damage and endothelial dysfunction are present in prediabetes as well, and a potential correlation of microvascular dysfunction with the risk of progression of prediabetes to DM and clinically overt CVD has been described [6].

A range of noninvasive methods has been developed to assess the microcirculation in patients with prediabetes across various vascular beds, including the retina, skin, kidney, and coronary microcirculation [6]. Among the most studied indices, it has been found that microalbuminuria (MAU) is prevalent in 9.3% of impaired fasting glucose (IFG) patients and in 11.0% of impaired glucose tolerance (IGT) patients [7]. Moreover, there is evidence that MAU in patients with prediabetes is a risk factor for DM development [8]. Furthermore, a higher augmentation index (AIx) in patients with prediabetes has been reported [9]. Microvascular retinal alterations have been described in patients with prediabetes as well, and emerging findings propose that these changes are also correlated with an increased risk of DM development [6].

Skin microcirculation assessment offers an easily accessible vascular bed for the evaluation of generalized microvascular function, as alterations in the skin’s small vessels have been correlated with organ damage in patients with CVD [10]. In prediabetes, the skin microcirculation has been investigated through the evaluation of the peak blood oxygen saturation (OxyP) after arterial occlusion [11] and the skin hyperemic response using a laser Doppler system [12]. Recently, laser speckle contrast analysis (LASCA) has been introduced as a technique for the assessment of the skin microcirculation [10,13]. To the best of our knowledge, no study has evaluated the skin microcirculation in patients with prediabetes using the LASCA technique. The subendocardial viability ratio (SEVR) is a functional marker of coronary microvascular function, reflecting the balance of the myocardial oxygen supply and demand [14]. One study utilized the SEVR for the evaluation of myocardial microvascular perfusion in patients with prediabetes; however, no comparison was made with normoglycemic individuals or patients with DM [15]. Most studies utilize one or two methods concurrently for assessing microvascular function in each population. Furthermore, while the mutual presence of prediabetes and microvascular damage is well established in the literature, the simultaneous evaluation of multiple vascular beds has not been implemented in patients with prediabetes. For instance, it has recently been found that the skin microcirculation is impaired in the prediabetic status; however, it is unknown whether this damage is associated with myocardial microvascular dysfunction [11]. To address this gap, we developed, to the best of our knowledge, for the first time, a study simultaneously assessing the skin microcirculation using LASCA and the myocardial microvascular function using the SEVR in patients with prediabetes. This study extends previous research by applying LASCA in prediabetes and by comparing the SEVRs across normoglycemic controls, prediabetes, and type 2 DM. Additionally, in our study, the albumin-to-creatinine ratio (ACR) and AIx were included as well-established markers of vascular dysfunction. Retinal vessel calibers were selected as quantitative markers of the retinal microvascular structure. In clinical practice, retinal microvascular involvement in patients with DM is evaluated through fundoscopic evaluation and the presence of diabetic retinopathy [16]. Thus, we incorporated the retinal vessel caliber measurements to evaluate whether these indices could identify early microvascular alterations not captured by conventional retinal assessment.

Based on the findings of large-scale cohort studies, microvascular dysfunction is already present in prediabetes, with the Maastricht study reporting impairment of skin and retinal microvascular indices [12], and the KoGES [8] and KORA [17] presenting renal microvascular involvement in patients with prediabetes. However, the above studies either examined these vascular beds using established markers or used novel methods of microvascular assessment but examined only a limited number of vascular indices.

Guidelines logically prioritize specific markers for therapeutic management and follow-up based on practicality, cost-effectiveness, feasibility, and evidence from large-scale studies. However, could the inclusion of novel, more sensitive markers, such as LASCA and the SEVR, which are not included in the current guidelines, help diagnose target organ damage (TOD) and assess the early CVD risk in patients with prediabetes?

The objective of this study was to evaluate the presence of microvascular alterations in various vascular beds, using both established and novel techniques of microcirculation assessment, in patients with prediabetes compared to normoglycemic controls and patients with DM. Το examine the net effect of glucose impairment on microvascular damage, this study included only a meticulous sample consisting of individuals without established CVD, thereby limiting confounding due to established CVD. In addition, associations between skin and myocardial microvascular function and CVD risk factors, as well as other distinct microvascular parameters, were investigated.

## 2. Materials and Methods

### 2.1. Participants

This is a cross-sectional observational study. Participants were recruited using a convenience-based approach from August 2020 to October 2023. Individuals were screened for eligibility based on the inclusion and exclusion criteria of this study. Recruitment was temporarily interrupted during the COVID-19 pandemic. Participants were categorized into three groups according to their glycemic status: normoglycemic, prediabetes, and type 2 DM. According to the American Diabetes Association (ADA) criteria, the diagnosis of DM was made if one of the following criteria was met: (1) an elevated fasting plasma glucose (FPG) of ≥126 mg/dL or elevated random plasma glucose of ≥200 mg/dL, confirmed by repeated testing on another day; (2) a 2 h plasma glucose level ≥ 200 mg/dL during a 75 g oral glucose tolerance test (OGTT); or (3) glycated hemoglobin (HbA1c) ≥ 6.5%. Respectively, prediabetes was defined by either (1) an FPG 100 mg/dL to 125 mg/dL (IFG); or (2) a 2 h plasma glucose level during a 75 g OGTT 140 mg/dL to 199 mg/dL (IGT); or (3) HbA1c 5.7–6.4% [18].

Patients with established CVD or other significant comorbidities, as well as type 1 DM, were excluded from this study. The normoglycemic group was defined as the control and consisted of individuals frequency-matched for age, sex, blood pressure (BP), and body mass index (BMI). All participants were over 18 years old. Written informed consent was obtained before study enrollment. The study protocol was conducted in accordance with the principles of the Declaration of Helsinki and approved by the Medical School of Aristotle University of Thessaloniki Bioethics Committee (Reference 6.360; Approval Date 29 July 2020) [19].

### 2.2. Clinical Assessment

All participants were asked to refrain from smoking or drinking coffee, tea, or alcohol for at least 4 h before they visited our laboratory. A detailed medical history, including current medication, was obtained, and a thorough physical examination was performed. BMI was calculated in kg/m^2^. Office BP was recorded, in the sitting position, using a validated oscillometric device (Microlife AG Swiss Corporation, Widnau, Switzerland) with the appropriate cuff size according to a standard protocol based on guidelines [20]. Hypertension was defined as office systolic BP (SBP) and/or diastolic BP (DBP) ≥ 140/90 mmHg and/or treatment with antihypertensive medication, according to the guidelines [20].

Blood samples were collected from all participants after an overnight fast. HbA1c was quantified using high-performance liquid chromatography (Hb NEXT, Lawrenceville, GA, USA, Menarini Diagnostics, Athens, Greece) in whole-blood samples. Plasma glucose levels and lipid profiles (total cholesterol, low-density lipoprotein, high-density lipoprotein, and triglycerides) were determined using routine laboratory techniques (Architect c16000, Abbott, Abbott Park, IL, USA).

### 2.3. Microvascular Assessment

#### 2.3.1. Assessment of Skin Microvascular Function

Cutaneous blood flow was recorded using a LASCA device (PeriCam PSINR System, Perimed, Järfälla, Sweden). All measurements were performed in the supine position under the standardized conditions mentioned in detail in our previous studies [21,22].

After a 20 min acclimatization period, recording of skin microvascular perfusion was initiated. In the present study, we used the post-occlusive reactive hyperemia (PORH) protocol. It represents a temporary increase in blood flow following the release of a previously occluded artery [23,24]. The PORH protocol consisted of three phases: (a) a 3 min baseline recording, (b) a 5 min period of brachial artery occlusion by inflating a pressure cuff at 250 mmHg to occlude skin blood perfusion, and (c) a 5 min reperfusion period after rapid deflation of the cuff [22]. Two circular skin sites [regions of interest (ROIs)], with a 10 mm radius each, were randomly chosen on the surface of the examined forearm. The average blood perfusion of the two ROIs per testing period was used in the analysis [24].

Data analysis was performed using the manufacturer’s software (PIMSoft, Perimed, Järfälla, Sweden), and the recorded blood flux was expressed in arbitrary perfusion units (PU). Results are described as the mean perfusion during the baseline period (baseline flux, PU), percentage decrease in perfusion from baseline to maximum occlusion (%), mean perfusion at the maximum response of the post-occlusive period (peak flux, PU), and percentage increase in perfusion from baseline to the maximum post-occlusive response (peak magnitude, %).

The baseline-to-peak percentage change was selected as the primary LASCA outcome because it reflects the relative hyperemic response and allows for a comparison of microvascular reactivities, and it is used in similar PORH protocols. Additional LASCA-derived parameters derived during the protocol (e.g., area under the curve) were not included in the final analysis.

#### 2.3.2. Assessment of Microvascular Myocardial Perfusion and Peripheral Vascular Disease State

The SEVR, also called the Buckberg index [25], is an indicator of myocardial perfusion and reflects the balance of the myocardial oxygen supply and demand equilibrium [14]. It is calculated as the ratio of the diastolic pressure time index (DPTI), which is indicative of myocardial supply, to the systolic pressure time index (SPTI), which corresponds to oxygen demands [14]. The SEVR was calculated via applanation tonometry of the radial artery using the SphygmoCor XCEL device (AtCor Medical Pty Ltd., Chicago, IL, USA). The measurements were performed in the supine position after a 15 min rest period, according to a standardized protocol. The average of two successive consecutive measurements was used in the analysis [26]. SEVR measurements were obtained under the same conditions in the same visit, with the LASCA and BP assessments. No participants were excluded due to poor-quality tonometry recordings.

The AΙx is the most widely used waveform parameter [27,28]. It was calculated using noninvasive pulse wave analysis via the SphygmoCor XCEL device (AtCor Medical Pty Ltd.) in the supine position under standardized conditions [26,29]. As the AIx is affected by the heart rate, for the analysis, the aortic AIx corrected to a heart rate of 75 bpm [AIx75] was used [26].

#### 2.3.3. Retinal Vessel Analysis

All participants underwent bilateral, digital fundus photography via a nonmydriatic digital fundus camera (NIDEK AFC-230/210, NIDEK, Fremont, CA, USA). Two photographs of each eye were obtained. All photographs were qualitatively evaluated by an experienced ophthalmologist to confirm the presence or absence of diabetic retinopathy. The photograph with the best quality was selected for the analysis and measurement of the retinal vessel diameter. Retinal vessel width measurements were performed by two trained staff members of our laboratory who were masked to the subject’s identity and glycemic status group assignment. Retinal vessel measurements were performed using custom semi-automated analysis software developed by the Foundation for Research and Technology-Hellas (FORTH, Heraklion, Greece), which applies automated retinal vessel segmentation and diameter measurement, based on a previously validated framework [30,31,32]. The intra- and interrater variabilities for the analyses of 20 retinal photographs were 0.823 and 0.798, respectively, as previously reported using the same methodology [31]. The central retinal artery equivalent (CRAE) and central retinal vein equivalent (CRVE) were automatically calculated using the Parr and Hubbard [30,31,33]. The arterio/venous ratio (AVR) was calculated as the CRAE/CRVE ratio.

#### 2.3.4. Assessment of Urinary Albumin Excretion

The ACR, measured in a spot urine sample, was used to estimate albuminuria. The ACR is recognized as an early marker of endothelial dysfunction and CVD [34,35]. According to the ADA guidelines, normal urine albumin excretion was defined as an ACR ≤ 30 mg/g [36].

### 2.4. Statistical Analysis and Sample Size Calculation

Statistical analysis was conducted using SPSS 29.0 (IBM SPSS Statistics for Windows, Version 29.0. Armonk, NY, USA: IBM Corp). Categorical variables were presented as percentages (frequencies) and were compared using the chi-square test. Continuous variables were assessed for normality using the Shapiro–Wilk test, along with standard scores for skewness and kurtosis, as well as respective histograms and normal Q-Q plots. Normally distributed parameters were expressed as mean ± standard deviation (SD), and non-normal as median and interquartile range (IQR). An analysis of variance (ANOVA) and a *t*-test were used to calculate the three- and two-group differences, respectively, for normally distributed variables. Post hoc pairwise comparisons were adjusted using the Bonferroni correction to account for multiple testing among the three groups. Variables with non-normal distribution were compared using the Kruskal–Wallis test for three-group comparisons, followed, when appropriate, by post hoc pairwise comparisons using Dunn’s test, while two-group comparisons were performed using the Mann–Whitney test. Statistical analyses were performed without individual matching, as matching was frequency-based.

Univariate linear regression was performed to identify potential correlations of the microvascular parameters and other characteristics of the participants. Multivariate linear regression was performed and included variables with a statistically significant correlation in the univariate analysis with the microvascular skin reactivity and SEVR, using a *p*-value ≤ 0.05 as a threshold to enter the model. Multicollinearity was assessed prior to multivariate model construction. A logarithmic transformation was applied to non-normally distributed variables. A *p*-value of <0.05 was considered statistically significant.

Limited prior data are available, which do not allow for a priori sample size estimation. In the literature, only one study has evaluated skin microcirculation function using LASCA in patients with type 1 DM [37]. Based on these data, a minimum of 57 participants (19 per group) are required to detect a statistically significant difference at an alpha level of 0.05 with 80% power. Sample size calculations were performed using G*Power Software (version 3.1.9.7) [38].

## 3. Results

### 3.1. Participants’ Characteristics

This study included 67 individuals: 22 controls, 24 subjects with prediabetes, and 21 with DM. The baseline demographic and clinical characteristics of the study participants are presented in Table 1. The median duration from DM diagnosis was 18 (2–90) months. Among the DM group, 71.5% were under treatment, and 28.5% were managed with lifestyle interventions. In more detail, 93.3% received metformin, 13.3% a glucagon-like peptide-1 agonist, and 13.3% pioglitazone, while 13.3% received a sodium-glucose cotransporter-2 inhibitor, 13.3% a dipeptidyl peptidase-4 inhibitor, and 20% were on insulin treatment. Among individuals with prediabetes, only one received metformin for prediabetes, while none of the controls were under antidiabetic medication. The qualitative analysis of retinal vessels demonstrated diabetic retinopathy in only one individual with DM.

### 3.2. Microvascular Characteristics

The skin, retinal, renal, peripheral vascular, and myocardial index values in the three different groups are presented in Table 2.

**Figure 1:**
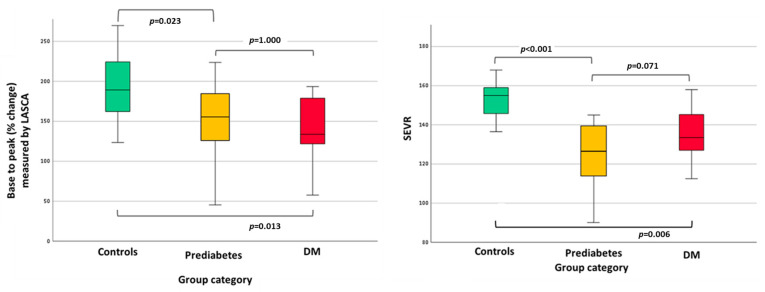
Skin and myocardial microcirculation assessment by glucose status group. Comparison of baseline-to-peak percentage changes following post-occlusive reactive hyperemia in controls, patients with prediabetes, and patients with DM, and myocardial oxygen consumption and supply, as evaluated by SEVR, in controls, patients with prediabetes, and patients with DM. DM, diabetes mellitus; LASCA, laser speckle contrast analysis; SEVR, subendocardial viability ratio.

Skin microcirculation assessment with LASCA revealed a statistically significant difference in the baseline flux between subjects with prediabetes and controls (*p* = 0.003). Participants with prediabetes and DM presented a significantly lower base-to-peak (% change) compared to controls (Figure 1). Furthermore, regarding the myocardial microcirculation, controls presented higher values of the SEVR compared to individuals with prediabetes and DM (Figure 1).

Evaluation of the renal microcirculation with the ACR, peripheral vessels with the AIx75, and retinal microvascular parameters (CRAE, CRVE, and AVR) did not show any statistically significant difference between the three groups.

### 3.3. Univariate Linear Regression

In the univariate analysis among all study individuals, the baseline-to-peak change correlated with the SEVR (r = 0.338, *p* = 0.017), age (r = −0.348, *p* = 0.004), office SBP (r = −0.278, *p* = 0.024), and glucose levels (r = −0.382, *p* = 0.002). The SEVR correlated with the heart rate (r = −0.562, *p* < 0.001) and AVR (r = −0.323, *p* = 0.045). The univariate analyses of the skin microvascular reactivity and SEVR in relation to the patient characteristics and other vascular parameters are presented in Supplemental Table S1.

### 3.4. Multivariate Linear Regression Analysis for Skin Microvascular Reactivity and Myocardial Perfusion in the Total Population

In the multivariate linear regression analysis (Table 3), the skin reactivity assessed by the baseline-to-peak (% change) was associated with the SBP levels and glucose levels. In comparison, the SEVR was associated with the office heart rate and AVR.

### 3.5. Subgroup Analysis in Patients Without Hypertension

To define the possible contribution of increased BP to the above results, subgroup analysis was performed in groups of controls and patients with prediabetes without hypertension. Only six participants with DM were normotensive; therefore, we decided to exclude the diabetic group from the present analysis. The subgroup analysis included 14 normotensive controls and 10 normotensive patients with prediabetes. The baseline characteristics of these participants are presented in Supplemental Table S2.

**Figure 2:**
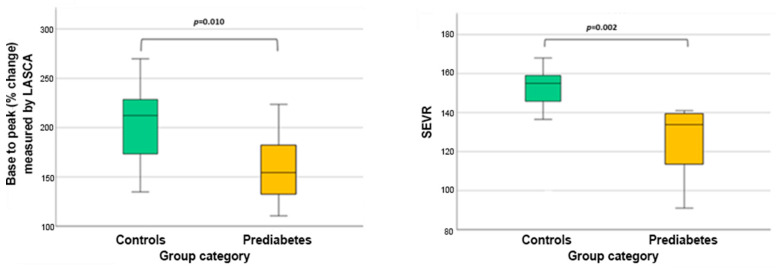
Comparison of baseline-to-peak percentage changes following post-occlusive reactive hyperemia in controls and patients with prediabetes without hypertension, and myocardial oxygen consumption and supply as evaluated by SEVR. LASCA, laser speckle contrast analysis; SEVR, subendocardial viability ratio.

In this analysis, patients with prediabetes, compared to controls, presented a decreased base-to-peak (% change) (Figure 2). Additionally, the SEVR was significantly higher in controls compared to that in patients with prediabetes (Figure 2). The microvascular indices of the participants of this subgroup are presented in Table 4.

## 4. Discussion

In the present study, we sought to explore the microvascular function across multiple vascular beds using established and novel (LASCA, SEVR) noninvasive microvascular assessment methods in a population from normoglycemia to DM without established CVD. For the first time, to the best of our knowledge, the skin microcirculation was evaluated using the LASCA technique in patients with prediabetes, showing that patients with prediabetes and DM exhibited significantly lower microvascular reactivity, compared to controls. These findings suggest that alterations in the skin microcirculation may already be present in the prediabetic status. To the best of our knowledge, this is also the first study to compare the myocardial microcirculation assessed by the SEVR between normoglycemic controls and patients with prediabetes and DM in the same cohort. The myocardial perfusion, as reflected by the SEVR, was lower in patients with prediabetes and those with DM compared to controls, while no significant statistical differences were identified between patients with prediabetes and those with DM. In contrast, the diabetic retinopathy signs, retinal vessel diameter assessments, and ACR did not vary among the three groups.

Furthermore, recognizing that high BP significantly contributes to micro- and macrovascular damage in patients with DM, a subgroup analysis of participants without hypertension revealed that the skin microcirculation and myocardial perfusion in patients with prediabetes remained attenuated when compared to that of the control group. The study design maintained consistent baseline characteristics across the three groups, except for antihypertensive medication, statin use, glucose, and HbA1c levels, enabling us to analyze the net glucose effect on these patients’ vascular networks.

### 4.1. Skin Microcirculatory Dysfunction in Prediabetes

Our findings suggest that the skin microcirculation, assessed with LASCA, was attenuated in patients with prediabetes and DM (based on the % change from base to peak) compared to that of controls. At the same time, no statistically significant difference was observed between patients with prediabetes and those with DM. We found that glucose levels were associated with an attenuated skin response, as assessed by LASCA. The skin microcirculation in patients with prediabetes has been evaluated with the OxyP technique in a large-population study. The OxyP was decreased in patients with prediabetes (86.8 ± 6) and DM (85.2 ± 6) compared to normoglycemic controls (88.2 ± 5.9) (*p* < 0.05 for all) [11], revealing, in accordance with our findings, evidence of altered skin microcirculation in patients with prediabetes. In the Maastricht study, the skin microcirculation was evaluated by measuring the skin’s hyperemic response using a laser Doppler system, and the %-hyperemia was reduced in prediabetes [β = −46, 95% confidence interval (CI): −163 to −72] and declined further in type 2 DM (β = −184, 95% CI: −297 to −71) compared to normoglycemia, showing a significant difference (*p* for all = 0.001) [12]. Similarly, our findings are consistent with those of others, showing that attenuated skin microcirculation has been independently associated with FPG (β = −0.10, 95% CI: −0.15 to −0.04) [12]. Hu et al., using a noninvasive technique that combines laser–Doppler flowmetry (LDF) with spectral (wavelet) analysis, investigated the skin microcirculation in patients with prediabetes and DM. There was a progressive decrease in the relative energy contribution from normal individuals to patients with prediabetes and DM [39].

We found that patients with prediabetes had higher baseline perfusion compared to controls. Although this observation is reported here for the first time in prediabetes, our group has found that an increased baseline flux compared to controls is also observed in other patients who are at increased cardiovascular risk, such as patients with hypertension [10] and patients with autoimmune disorders [22]. We speculated that the unexpected increase in the baseline flux may reflect the compensatory recruitment of additional functional small vessels in response to early microvascular damage. However, this interpretation remains hypothetical and cannot be confirmed by the cross-sectional design of this study.

### 4.2. Myocardial Microvascular Dysfunction in Prediabetes

Our study showed that the microvascular myocardial perfusion assessed by the SEVR was lower in patients with prediabetes and those with DM compared to that of controls, but no difference was observed between patients with prediabetes and those with DM, while the SEVR remained reduced when a subgroup analysis was performed, excluding patients with hypertension. These findings suggest that alterations in myocardial microvascular perfusion may occur early in the course of dysglycemia. As far as we are aware, one study to date has used the SEVR to assess the myocardial perfusion in 230 individuals with prediabetes. In the study, patients with prediabetes with higher estimated glomerular filtration rates (eGFRs) had lower SEVRs, and the SEVR was associated with the SBP, HOMA-IR, and eGFR [15]. However, that study did not include normoglycemic controls or patients with DM, limiting direct comparisons across different glycemic states.

### 4.3. Renal Microvascular Alterations in Prediabetes

Our results showed that the ACR did not differ significantly across the three groups. This finding may be attributed to the relatively good glycemic control in the cohort of patients with DM and the relatively short duration from DM diagnosis. Although multiple population-based studies have reported associations between prediabetes and increased ACR, results across the literature are not fully consistent. Several large-population-based studies have reported that a positive association exists between increased ACR and blood glucose levels or prediabetes, along with a higher future risk for the development of DM [8,17,40]

In contrast, other cohorts have reported that prediabetes is not independently associated with microalbuminuria [41,42]. It is well established that the urinary ACR is an early indicator of endothelial dysfunction, regardless of blood glucose levels [34]. Differences in study designs, population characteristics, and disease durations may explain these differences. In this context, the absence of differences in the ACR in our cohort, which was characterized by the absence of CVD, reflects an earlier disease stage, before overt renal microvascular disease becomes detectable.

### 4.4. Retinal Microvascular Indices in Prediabetes

Only one patient with DM and none of the patients with prediabetes had signs of diabetic retinopathy. This finding reflects that patients with DM had well-controlled DM and a relevant recent DM diagnosis, as well as that this index cannot be easily used to identify the TOD in most individuals with prediabetes. To overcome this, we additionally studied more subtle microvascular indices by evaluating the retinal vessel diameters. However, no differences were observed in the CRAE, CRVE, and AVR, even after a subgroup analysis was performed in participants without hypertension. In a study of 2005 participants, including patients with prediabetes, those with DM, and normoglycemic individuals, prediabetes was associated with macular thinning and a reduced CRAE [43]. According to findings from the Rotterdam study, prediabetes was associated with a larger diameter of the retinal vein in individuals with IFG status [44]. However, in all the above studies, hypertension was not excluded as a confounding factor. In this context, the absence of differences in the retinal vessel diameters in our CVD-free cohort, including a significant proportion of normotensive individuals, may reflect an earlier disease stage, where structural retinal microvascular changes may not yet be apparent.

### 4.5. Peripheral Vascular Function in Prediabetes

According to our findings, the AIx did not present any statistical significance across the three groups. There is evidence of increased AIx levels in adults with prediabetes compared to normoglycemic controls [9,45]. However, in accordance with our findings, in a study conducted by Shah and colleagues, the AIx levels did not differ significantly across the normoglycemic participants and patients with prediabetes [46].

The absence of differences in these established indices (retinal, ACR, and AIx) may be related to the relatively short duration of DM and the generally good glycemic control in our cohort, which may have limited the development of detectable microvascular changes.

### 4.6. Strengths and Limitations

A significant strength of this study is that the participants were meticulously selected (without established CVD, with no differences in age, BMI, and BP levels, and with a relatively short duration and good glycemic control for DM), diminishing potential confounding factors. Additionally, an extensive assessment of the microvascular function was performed in all study participants, whereas most studies to date have used only one or two methods for assessing microvascular dysfunction. The methods we used for microvascular evaluation were noninvasive and feasible, allowing for the exploration of microvascular alterations across multiple vascular beds in a research setting.

Despite the interesting results, our study has some limitations. The relatively small sample size limits the potential for examining risk factors. The cross-sectional design of this study confines the establishment of causality between the disease and potential risk factors. In the future, large prospective studies could provide further insights into the role of novel microvascular assessment techniques (LASCA, SEVR) as potentially useful tools in risk stratification and treatment decisions in patients with prediabetes. Moreover, differences in the antihypertensive medication and statin use between groups may have influenced vascular function and should be considered as a potential source of residual confounding [47,48]. However, the BP and serum lipid levels were comparable across the three study groups, which may have limited the impact of these therapies on vascular measurements. It is well known that patients with DM have increased BP and serum lipid levels; achieving similar BP levels and lipid profiles in this cohort required more frequent use of antihypertensive and lipid-lowering medications in patients with DM. Although antihypertensive treatment was included in the multivariate model (for LASCA), differences in statin or antidiabetic medication use between groups were not included, as only variables significant in univariate analyses (*p* ≤ 0.05) were entered into the multivariate models due to the small sample size. Thus, sensitivity analyses excluding patients receiving these medications were not performed.

## 5. Conclusions

In this study, a comprehensive assessment of the microvascular circulation utilizing five conventional and novel techniques was performed in individuals with prediabetes and type 2 DM and normoglycemic controls. Assessment of the renal microcirculation with the ACR, peripheral vessels with the AIx75, and retinal microvascular parameters (CRAE, CRVE, and AVR) did not show any statistically significant difference across the three groups. However, LASCA provided access to the skin microcirculation, which was found to be lower in this group and similar to that in the patients with DM. Assessment of myocardial perfusion using the SEVR showed lower values in patients with prediabetes and DM compared with normoglycemic controls. Our findings suggest that LASCA and the SEVR may serve as sensitive markers for the detection of early, subclinical microvascular dysfunction in patients with prediabetes.

Glucose levels, SBP, and age were inversely correlated with the base-to-peak change assessed by LASCA, potentially reflecting their role in the skin microcirculation impairment. Baseline-to-peak (% change) and SEVR levels were positively correlated, suggesting a possible crosstalk between these vascular beds. In multivariate analysis, SBP and glucose were independently associated with skin microvascular dysfunction, while the heart rate and arteriovenous ratio were associated with the SEVR. Moreover, a subgroup analysis was performed among normoglycemic controls and patients with prediabetes without hypertension, showing that the skin microcirculation and myocardial perfusion remained lower in this subgroup. Larger studies are needed to confirm these results and further explore the clinical significance of early microvascular changes in both prediabetes and DM.

## Figures and Tables

**Table 1:** Baseline characteristics of all participants.

	Total, n = 67	Controls, n = 22	Prediabetes, n = 24	Type 2 Diabetes, n = 21	*p*-Value
Gender (male) % (n)	43.3 (29)	40.9 (9)	37.5 (9)	52.4 (11)	0.581
Age (years)	55.9 ± 9.4	53.2 ± 8.6	55.6 ± 10.9	59.1 ± 7.7	0.113
Hypertension (yes) % (n)	55.2 (37)	36.4 (8)	58.3 (14)	71.4 (15)	0.064
Smoking % (n) Current Past	41.5 (27) 13.8 (9)	36.4 (8) 4.5 (1)	45.8 (11) 16.7 (4)	42.1 (8) 21.1 (4)	0.401
BMI (kg/m^2^)	29.4 ± 5.7	27.9 ± 5.8	30.2 ± 6	30.1 ± 5.2	0.340
Glucose (mg/dL)	99 (90.3–118.8)	89.5 (85.8–95)	103 (95–127.4)	120.4 (104–133)	<0.001 *^,+,‡^
HbA1c_%_	5.9 (5.5–6.2)	5.4 (5.2–5.6)	5.8 (5.6–6)	6.2 (5.9–6.7)	<0.001 *^,+,‡^
eGFR (mL/min/1.73 m^2^)	82.6 ± 17.1	88.6 ± 15.7	80.8 ± 13.8	78.2 ± 20.2	0.114
Total cholesterol (mg/dL)	198.8 ± 43.9	205.5 ± 38.1	208.6 ± 38.6	180.6 ± 51.2	0.084
LDL cholesterol (mg/dL)	123 (102–137)	119 (109.5–157.5)	124 (108–148.9)	109.4 (88.3–131.2)	0.221
Office SBP (mmHg)	129.6 ± 16.2	126.8 ± 18.1	129.9 ± 12.4	132.1 ± 18.1	0.567
Office DBP (mmHg)	80.5 ± 9.5	82.3 ± 11.9	78.4 ± 7.7	81.1 ± 8.4	0.362
Office heart rate (pulses/min)	72.9 ± 10.4	69.8 ± 9.3	75.5 ± 12.2	73.5 ± 9.0	0.201
Skin temperature levels (°C)	35.7 ± 0.3	35.9 ± 0.2	36 ± 0.3	36 ± 0.4	0.649
Under antihypertensive medication % (n)	41.8 (28)	18.2 (4)	41.7 (10)	66.7 (14)	0.006 ^‡^
Under therapy with statin % (n)	25.8 (17)	9.1 (2)	12.5 (3)	60.0 (12)	<0.001 ^+,‡^

Statistically significant differences between subgroups: * control–prediabetes, ^+^ prediabetes–diabetes, ^‡^ control–diabetes; BMI, body mass index; DBP, diastolic blood pressure; eGFR, estimated glomerular filtration rate; LDL, low-density lipoprotein; SBP, systolic blood pressure. Continuous variables are presented as mean ± standard deviation or as median (interquartile range), according to the distribution of the variable.

**Table 2:** Microvascular characteristics of study population.

	Total	Controls	Prediabetes	Type 2 Diabetes	*p*-Value
Baseline mean perfusion (baseline flux, PU)	43.7 ± 11.5	38.7 ± 8.5	49.6 ± 11.3	42 ± 12.0	0.003 *
Baseline-to-occlusion (% change)	−80.4 ± 8.0	−79.4 ± 9.7	−80.6 ± 8	−81.2 ± 6.0	0.769
Baseline-to-peak (% change)	163.3 ± 53.6	192.1 ± 42.4	151.2 ± 46.4	146.2 ± 61.4	0.006 *^,‡^
ACR (mg/g)	6.5 (2.9–11.3)	5.7 (5.1–11.8)	5 (0.5–12.1)	7.8 (6.8–12.4)	0.125
CRAE (μm)	89 ± 11.5	87.4 ± 14.2	91.4 ± 9.1	88.1 ± 11.2	0.586
CRVE (μm)	114.3 ± 17.5	117.1 ± 16.5	111 ± 18.8	115.5 ± 20.5	0.562
AVR	0.8 ± 0.2	0.7 ± 0.15	0.8 ± 0.2	0.8 ± 0.2	0.224
SEVR	133.6 (118.9–146.1)	155 (142.5–160)	127.5 (114.1–139.4)	133.5 (124–145.5)	0.001 *^,‡^
AIx75	31.9 ± 14.1	31 ± 8.5	34.6 ± 18.9	29.4 ± 10.4	0.467

Statistically significant differences between subgroups: * control–prediabetes, ^‡^ control–diabetes; ACR, albumin/creatinine ratio; AIx75, augmentation index adjusted to heart rate of 75 beats per minute; AVR, arteriovenous ratio; CRAE, central retinal arteriolar equivalent; CRVE, central retinal venular equivalent; SEVR, subendocardial viability ratio. Continuous variables are presented as mean ± standard deviation or as median (interquartile range), according to the distribution of the variable.

**Table 3:** Multivariate linear regression analysis for skin microvascular reactivity and myocardial perfusion in the total population.

Variables	B (Unstandardized Coefficient)	95% CI	*p*-Value
1. Dependent Variable: LASCA—baseline-to-peak (%change)			
Adjusted R^2^ = 0.327			
Age (years)	−0.362	−4.053 to 0.054	0.056
Office SBP (mmHg)	−0.297	−2.347 to −0.044	0.042
SEVR	0.198	−0.233 to 1.327	0.164
Glucose levels (mg/dL)	−0.185	−0.300 to −0.010	0.038
Antihypertensive drug use (yes/no)	−0.091	−55.653 to 34.613	0.640
2. Dependent Variable: SEVR			
Adjusted R^2^ = 0.429			
AVR	−0.306	−69.963 to −3.588	0.031
Office heart rate (beats per minute)	−0.526	−1.536 to −0.475	<0.001
Baseline-to-peak (% change)	0.142	−0.053 to 0.160	0.316

AVR, arteriovenous ratio; CI, confidence interval; LASCA, laser speckle contrast analysis; SBP, systolic blood pressure; SEVR, subendocardial viability ratio.

**Table 4:** Microvascular characteristics of participants without hypertension, stratified by glycemic status.

	Controls (n = 14)	Prediabetes (n = 10)	*p*-Value
Baseline mean perfusion (baseline flux, PU)	43.8 ± 6.3	46.1 ± 8.8	0.980
Baseline-to-occlusion (% change)	−81.8 ± 6.2	−83.5 ± 7.7	0.194
Peak perfusion	120.1 (103.1–133.2)	113.9 (106.1–123.6)	0.859
Baseline-to-peak (% change)	194.9 ± 39.3	160.9 ± 36.9	0.010
ACR (mg/g)	5.7 (5.4–9.6)	1 (0.25–12.9)	0.129
CRAE (μm)	87.1 ± 15.3	89.7 ± 8.5	0.685
CRVE (μm)	116 ± 10.0	115.8 ± 15.6	0.871
AVR	0.8 ± 0.1	0.8 ± 0.1	0.525
SEVR	155 (142.5–160)	133.3 (112.6–139.5)	0.002
AIx75	33 (23.5–38.5)	37.5 (21.5–49.3)	0.476

ACR, albumin/creatinine ratio; AIx75, augmentation index adjusted to heart rate of 75 beats per minute; AVR, arteriovenous ratio; CRAE, central retinal arteriolar equivalent; CRVE, central retinal venular equivalent; SEVR, subendocardial viability ratio. Continuous variables are presented as mean ± standard deviation or as median (interquartile range), according to the distribution of the variable.

## Data Availability

The data are available upon reasonable request to the corresponding author.

## Supplementary Materials

The following supporting information can be downloaded at: https://www.mdpi.com/article/10.3390/life16020326/s1, Table S1: Correlations of Skin and Myocardial Microvascular Function with Patient Characteristics and Vascular Parameters in the Total Study Population; Table S2: Baseline characteristics of participants without arterial hypertension.

## Abbreviations

ACR	Albumin-to-creatinine ratio
AIx	Augmentation index
AIx75	Augmentation index corrected to a heart rate of 75 bpm
ARIC	Atherosclerosis Risk in Communities
AVR	Arterio/venous ratio
CMI	Cardio-metabolic index
CVD	Cardiovascular disease
CRAE	Central retinal artery equivalent
CRVE	Central retinal vein equivalent
DBP	Diastolic blood pressure
DM	Diabetes mellitus
DPTI	Diastolic pressure time index
MAU	Microalbuminuria
FPG	Fasting plasma glucose
IFG	Impaired fasting glucose
IGT	Impaired glucose tolerance
LASCA	Laser speckle contrast analysis
LDF	Laser–Doppler flowmetry
OxyP	Peak blood oxygen saturation
PORH	Post-occlusive reactive hyperemia
PU	Perfusion units
ROIs	Regions of interest
SBP	Systolic blood pressure
SPTI	Systolic pressure time index
SEVR	Subendocardial viability ratio
TOD	Target organ damage

## References

[1] The Emerging Risk Factors Collaboration (2011). Diabetes Mellitus, Fasting Glucose, and Risk of Cause-Specific Death. N. Engl. J. Med..

[2] Raghavan S., Vassy J.L., Ho Y., Song R.J., Gagnon D.R., Cho K., Wilson P.W.F., Phillips L.S. (2019). Diabetes Mellitus–Related All-Cause and Cardiovascular Mortality in a National Cohort of Adults. J. Am. Heart Assoc..

[3] Sarwar N., Gao P., Seshasai S.R.K., Gobin R., Kaptoge S., Di Angelantonio E., Ingelsson E., Lawlor D.A., Selvin E., Emerging Risk Factors Collaboration (2010). Diabetes Mellitus, Fasting Blood Glucose Concentration, and Risk of Vascular Disease: A Collaborative Meta-Analysis of 102 Prospective Studies. Lancet.

[4] Rooney M.R., He J.H., Salpea P., Genitsaridi I., Magliano D.J., Boyko E.J., Wallace A.S., Fang M., Selvin E. (2025). Global and Regional Prediabetes Prevalence: Updates for 2024 and Projections for 2050. Diabetes Care.

[5] Cai X., Zhang Y., Li M., Wu J.H., Mai L., Li J., Yang Y., Hu Y., Huang Y. (2020). Association between Prediabetes and Risk of All Cause Mortality and Cardiovascular Disease: Updated Meta-Analysis. BMJ.

[6] Lamprou S., Koletsos N., Mintziori G., Anyfanti P., Trakatelli C., Kotsis V., Gkaliagkousi E., Triantafyllou A. (2023). Microvascular and Endothelial Dysfunction in Prediabetes. Life.

[7] Tapp R.J., Shaw J.E., Zimmet P.Z., Balkau B., Chadban S.J., Tonkin A.M., Welborn T.A., Atkins R.C. (2004). Albuminuria Is Evident in the Early Stages of Diabetes Onset: Results from the Australian Diabetes, Obesity, and Lifestyle Study (AusDiab). Am. J. Kidney Dis..

[8] Jung D.-H., Byun Y.-S., Kwon Y.-J., Kim G.-S. (2017). Microalbuminuria as a Simple Predictor of Incident Diabetes over 8 Years in the Korean Genome and Epidemiology Study (KoGES). Sci. Rep..

[9] Di Pino A., Scicali R., Calanna S., Urbano F., Mantegna C., Rabuazzo A.M., Purrello F., Piro S. (2014). Cardiovascular Risk Profile in Subjects with Prediabetes and New-Onset Type 2 Diabetes Identified by HbA(1c) According to American Diabetes Association Criteria. Diabetes Care.

[10] Lazaridis A., Triantafyllou A., Mastrogiannis K., Malliora A., Doumas M., Gkaliagkousi E. (2023). Assessing Skin Microcirculation in Patients at Cardiovascular Risk by Using Laser Speckle Contrast Imaging. A Narrative Review. Clin. Physiol. Funct. Imaging.

[11] Cederqvist J., Rådholm K., Nystrom F.H., Engvall J., Bergstrand S., Fredriksson I., Strömberg T., Östgren C.J. (2025). Impaired Microcirculation in the Skin and Subclinical Atherosclerosis in Individuals with Dysglycaemia in a Large Population-Based Cohort. Cardiovasc. Diabetol..

[12] Sörensen B.M., Houben A.J.H.M., Berendschot T.T.J.M., Schouten J.S.A.G., Kroon A.A., van der Kallen C.J.H., Henry R.M.A., Koster A., Sep S.J.S., Dagnelie P.C. (2016). Prediabetes and Type 2 Diabetes Are Associated with Generalized Microvascular Dysfunction: The Maastricht Study. Circulation.

[13] Senarathna J., Rege A., Li N., Thakor N. (2013). V Laser Speckle Contrast Imaging: Theory, Instrumentation and Applications. IEEE Rev. Biomed. Eng..

[14] Xie H., Gao L., Fan F., Gong Y., Zhang Y. (2024). Research Progress and Clinical Value of Subendocardial Viability Ratio. J. Am. Heart Assoc..

[15] Di Pino A., Scicali R., Marchisello S., Zanoli L., Ferrara V., Urbano F., Filippello A., Di Mauro S., Scamporrino A., Piro S. (2021). High Glomerular Filtration Rate Is Associated with Impaired Arterial Stiffness and Subendocardial Viability Ratio in Prediabetic Subjects. Nutr. Metab. Cardiovasc. Dis..

[16] American Diabetes Association Professional Practice Committee for Diabetes (2026). 12. Retinopathy, Neuropathy, and Foot Care: Standards of Care in Diabetes—2026. Diabetes Care.

[17] Markus M.R.P., Ittermann T., Baumeister S.E., Huth C., Thorand B., Herder C., Roden M., Siewert-Markus U., Rathmann W., Koenig W. (2018). Prediabetes Is Associated with Microalbuminuria, Reduced Kidney Function and Chronic Kidney Disease in the General Population: The KORA (Cooperative Health Research in the Augsburg Region) F4-Study. Nutr. Metab. Cardiovasc. Dis..

[18] American Diabetes Association (2021). 2. Classification and Diagnosis of Diabetes: Standards of Medical Care in Diabetes—2021. Diabetes Care.

[19] World Medical Association (2013). World Medical Association Declaration of Helsinki: Ethical Principles for Medical Research Involving Human Subjects. JAMA.

[20] Mancia G., Kreutz R., Brunström M., Burnier M., Grassi G., Januszewicz A., Muiesan M.L., Tsioufis K., Agabiti-Rosei E., Algharably E.A.E. (2023). 2023 ESH Guidelines for the Management of Arterial Hypertension the Task Force for the Management of Arterial Hypertension of the European Society of Hypertension. J. Hypertens..

[21] Lamprou S., Koletsos N., Zografou I., Lazaridis A., Mintziori G., Trakatelli C.M., Kotsis V., Gkaliagkousi E., Doumas M., Triantafyllou A. (2024). Skin Microvascular Dysfunction in Type 2 Diabetes Mellitus Using Laser Speckle Contrast Analysis and Association with Carotid Intima-Media Thickness. J. Clin. Med..

[22] Koletsos N., Gkaliagkousi E., Lazaridis A., Triantafyllou A., Anyfanti P., Dolgyras P., Dipla K., Galanopoulou V., Aslanidis S., Douma S. (2021). Skin Microvascular Dysfunction in Systemic Lupus Erythematosus Patients with and without Cardiovascular Risk Factors. Rheumatology.

[23] Roustit M., Cracowski J.-L. (2012). Non-Invasive Assessment of Skin Microvascular Function in Humans: An Insight into Methods. Microcirculation.

[24] Roustit M., Cracowski J.-L. (2013). Assessment of Endothelial and Neurovascular Function in Human Skin Microcirculation. Trends Pharmacol. Sci..

[25] Buckberg G.D., Fixler D.E., Archie J.P., Hoffman J.I. (1972). Experimental Subendocardial Ischemia in Dogs with Normal Coronary Arteries. Circ. Res..

[26] Laurent S., Cockcroft J., Van Bortel L., Boutouyrie P., Giannattasio C., Hayoz D., Pannier B., Vlachopoulos C., Wilkinson I., Struijker-Boudier H. (2006). Expert Consensus Document on Arterial Stiffness: Methodological Issues and Clinical Applications. Eur. Heart J..

[27] Azizzadeh M., Karimi A., Breyer-Kohansal R., Hartl S., Breyer M.-K., Gross C., Boutouyrie P., Bruno R.M., Hametner B., Wassertheurer S. (2024). Reference Equations for Pulse Wave Velocity, Augmentation Index, Amplitude of Forward and Backward Wave in a European General Adult Population. Sci. Rep..

[28] Westerhof N., Westerhof B.E. (2017). Waves and Windkessels Reviewed. Artery Res..

[29] Kesten S., Qasem A., Avolio A. (2022). Viewpoint: The Case for Non-Invasive Central Aortic Pressure Monitoring in the Management of Hypertension. Artery Res..

[30] Manikis G.C., Sakkalis V., Zabulis X., Karamaounas P., Triantafyllou A., Douma S., Zamboulis C., Marias K. (2011). An Image Analysis Framework for the Early Assessment of Hypertensive Retinopathy Signs. Proceedings of the 2011 E-Health and Bioengineering Conference (EHB).

[31] Triantafyllou A., Doumas M., Anyfanti P., Gkaliagkousi E., Zabulis X., Petidis K., Gavriilaki E., Karamaounas P., Gkolias V., Pyrpasopoulou A. (2013). Divergent Retinal Vascular Abnormalities in Normotensive Persons and Patients with Never-Treated, Masked, White Coat Hypertension. Am. J. Hypertens..

[32] Triantafyllou A., Anyfanti P., Gavriilaki E., Zabulis X., Gkaliagkousi E., Petidis K., Triantafyllou G., Gkolias V., Pyrpasopoulou A., Douma S. (2014). Association between Retinal Vessel Caliber and Arterial Stiffness in a Population Comprised of Normotensive to Early-Stage Hypertensive Individuals. Am. J. Hypertens..

[33] Hubbard L.D., Brothers R.J., King W.N., Clegg L.X., Klein R., Cooper L.S., Sharrett A.R., Davis M.D., Cai J. (1999). Methods for Evaluation of Retinal Microvascular Abnormalities Associated with Hypertension/Sclerosis in the Atherosclerosis Risk in Communities Study. Ophthalmology.

[34] Bartz S.K., Caldas M.C., Tomsa A., Krishnamurthy R., Bacha F. (2015). Urine Albumin-to-Creatinine Ratio: A Marker of Early Endothelial Dysfunction in Youth. J. Clin. Endocrinol. Metab..

[35] Kakutani Y., Morioka T., Mori K., Yamazaki Y., Ochi A., Kurajoh M., Fukumoto S., Shioi A., Shoji T., Inaba M. (2020). Albuminuria Rather than Glomerular Filtration Rate Is Associated with Vascular Endothelial Function in Patients with Type 2 Diabetes. J. Diabetes Complicat..

[36] American Diabetes Association (2021). 11. Microvascular Complications and Foot Care: Standards of Medical Care in Diabetes-2021. Diabetes Care.

[37] de M Matheus A.S., Clemente E.L.S., de Lourdes Guimarães Rodrigues M., Torres Valença D.C., Gomes M.B. (2017). Assessment of Microvascular Endothelial Function in Type 1 Diabetes Using Laser Speckle Contrast Imaging. J. Diabetes Complicat..

[38] Faul F., Erdfelder E., Buchner A., Lang A.-G. (2009). Statistical Power Analyses Using G*Power 3.1: Tests for Correlation and Regression Analyses. Behav. Res. Methods.

[39] Hu H.-F., Hsiu H., Sung C.-J., Lee C.-H. (2017). Combining Laser-Doppler Flowmetry Measurements with Spectral Analysis to Study Different Microcirculatory Effects in Human Prediabetic and Diabetic Subjects. Lasers Med. Sci..

[40] Melsom T., Schei J., Stefansson V.T.N., Solbu M.D., Jenssen T.G., Mathisen U.D., Wilsgaard T., Eriksen B.O. (2016). Prediabetes and Risk of Glomerular Hyperfiltration and Albuminuria in the General Nondiabetic Population: A Prospective Cohort Study. Am. J. Kidney Dis..

[41] Friedman A.N., Marrero D., Ma Y., Ackermann R., Narayan K.M.V., Barrett-Connor E., Watson K., Knowler W.C., Horton E.S. (2008). Diabetes Prevention Program Research Group Value of Urinary Albumin-to-Creatinine Ratio as a Predictor of Type 2 Diabetes in Pre-Diabetic Individuals. Diabetes Care.

[42] Kim C.-H., Kim K.-J., Kim B.-Y., Jung C.-H., Mok J.-O., Kang S.-K., Kim H.-K. (2014). Prediabetes Is Not Independently Associated with Microalbuminuria in Korean General Population: The Korea National Health and Nutrition Examination Survey 2011–2012 (KNHANES V-2,3). Diabetes Res. Clin. Pract..

[43] Huru J., Leiviskä I., Saarela V., Liinamaa M.J. (2021). Prediabetes Influences the Structure of the Macula: Thinning of the Macula in the Northern Finland Birth Cohort. Br. J. Ophthalmol..

[44] Ikram M.K., Janssen J.A.M.J.L., Roos A.M.E., Rietveld I., Witteman J.C.M., Breteler M.M.B., Hofman A., van Duijn C.M., de Jong P.T.V.M. (2006). Retinal Vessel Diameters and Risk of Impaired Fasting Glucose or Diabetes: The Rotterdam Study. Diabetes.

[45] Di Marco M., Scilletta S., Miano N., Capuccio S., Musmeci M., Di Mauro S., Filippello A., Scamporrino A., Bosco G., Di Giacomo Barbagallo F. (2025). Triglycerides to High Density Lipoprotein Cholesterol Ratio (TG/HDL), but Not Triglycerides and Glucose Product (TyG) Index, Is Associated with Arterial Stiffness in Prediabetes. Diabetes Res. Clin. Pract..

[46] Shah A.S., Gao Z., Urbina E.M., Kimball T.R., Dolan L.M. (2014). Prediabetes: The Effects on Arterial Thickness and Stiffness in Obese Youth. J. Clin. Endocrinol. Metab..

[47] Cavero-Redondo I., Saz-Lara A., Lugones-Sánchez C., Pozuelo-Carrascosa D.P., Gómez-Sánchez L., López-Gil J.F., García-Ortiz L., Bruno R.M., Gómez-Marcos M.Á. (2023). Comparative Effect of Antihypertensive Drugs in Improving Arterial Stiffness in Adults with Hypertension (RIGIPREV Study). A Network Meta-Analysis. Front. Pharmacol..

[48] Gepner A.D., Lazar K., Van Hulle C., Korcarz C.E., Asthana S., Carlsson C.M. (2019). Effects of Simvastatin on Augmentation Index Are Transient: Outcomes From a Randomized Controlled Trial. J. Am. Heart Assoc..

