# Supplementary material for: Microvascular Dysfunction in Patients with Prediabetes: Novel Methods Identify Impaired Microcirculation
Source: Life (Basel). 2026 Feb 13;16(2):326. doi: 10.3390/life16020326 (PMC12941821; doi:10.3390/life16020326)
Supplement: Supplementary file 1 [file life-16-00326-s001.zip › life-4151001-supplementary.pdf]

Supplemental Table 1a. Correlation coefficients between skin microvascular reactivity (assessed by base-to-peak % change) and ~~with~~ other patients’ characteristics and vascular parameters in ~~the~~ total study population.

	Correlation C~~c~~oefficient	p-value	95% Confidence Interval (2-tailed)	
			Lower	Upper
Gender	-.017	.895	-.258	.226
Age	-.348	.004	-.544	-.115
Office SBP	-.278	.024	-.487	-.039
Office DBP	-.103	.408	-.337	.142
Office heart rate	-.212	.095	-.437	.037
Antihypertensive medication	.257	.037	.016	.470
Hypertension	.273	.027	.033	.483
Statins	-.086	.794	-.323	.161
Smoking	.152	.231	-.098	.383
BMI	-.049	.701	-.289	.198
CRAE	-.244	.095	-.494	.043
CRVE	.029	.839	-.244	.297
AVR	-.177	.229	-.439	.113
AIx75	-.049	.713	-.301	.210
SEVR	.338	.017	.063	.566
Total cholesterol	-.201	.117	-.429	.051
eGFR	.173	.187	-0.085	.409
ACR	-.080	.589	-.363	.217
HbA1c	-.070	.597	-.325	.195
Glucose	-.382	.002	-.580	-.141
LDL	-.011	.935	-.267	.247

Supplemental Table 1b. Correlation coefficients between myocardial microvascular function (assessed by SEVR) and ~~with~~ other patients’ characteristics and vascular parameters in ~~the~~ total study population.

Sex	.002	.990	-.277	.280
Age	-.262	.066	-.504	.017
Office SBP	-.109	.452	-.376	.175
Office DBP	.019	.895	-.261	.296
Office HR	-.562	<.001	-.730	-.331
Hypertensive medication	.089	.541	-.195	.358
Hypertension	.159	.269	-.124	.419
Smoking	.063	.668	-.222	.338
Statins	-.004	.981	-.284	.278
BMI	-.148	.310	-.412	.139
Baseline (mean perfusion)	-.456	<.001	-.654	-.201
Peak perfusion	-.193	.184	-.450	.093
Base-to-occlusion (% change)	.057	.697	-.228	.333
Base-to-peak (% change)	.338	.017	.063	.566
CRAE	-.315	.051	-.573	.001
CRVE	.124	.421	-.179	.406
AVR	-.323	.045	-.579	-.008
AIx75	-.193	.179	-.447	.090
eGFR	.087	.576	-.216	.374
Total cholesterol	-.138	.361	-.412	.159
ACR	.305	.056	-.017	.569
Glucose	-.283	.051	-.531	.010
HbA1c	-.016	.915	-.313	.284

LDL	-.007	.963	-.305	.292

ACR, albumin/creatinine ratio; AIx75, augmentation index adjusted to ~~a~~ heart rate of 75 beats per minute; AVR, arteriovenous ratio; BMI, body mass index; BP, blood pressure; CRAE, central retinal arteriolar equivalent; CRVE, central retinal venular equivalent; DBP, diastolic blood pressure; eGFR, estimated glomerular filtration rate; Hb1Ac, hemoglobin A1c; LDL, low-density lipoprotein; SBP, systolic blood pressure; SEVR, subendocardial viability ratio.

Supplemental Table 2. Baseline characteristics of participants without arterial hypertension (controls and patients with prediabetes~~ies~~).

	Controls, n = 14	Prediabetes, n = 10	p-value
Gender (m~~M~~ale) % (n)	14.3 (2)	50 (5)	0.058
Age (years)	49.4 ±8.5	49.2 ±9.6	0.951
Under therapy with statin	14.3 (2)	0	0.212
BMI (kg/m^2)	27 (22.3- 31.8)	25.3 (23.6- 31.1)	0.886
Glucose (mg/dL)	89.5 (86.5- 95)	112 (98- 121.5)	<0.001
Hb1Ac%	5.3 ±0.2	5.9 ±0.4	0.006
eGFR	88.2 ±14.9	83.1 ±7.4	0.419
Total c~~C~~holesterol (mg/dL)	202.2 ±27.2	213.3 ±35.6	0.429
LDL c~~C~~holesterol (mg/dL)	125.3 ±32.9	133.8 ±22.9	0.53
Office SBP(mmHg)	118.9 ±10.2	123.5 ±9.4	0.274
Office DBP (mmHg)	76.5 ±7.4	78.1 ±5.2	0.571
Office heart rate (pulses/min)	67.4 ±9.8	80.3 ±10.1	0.005

BMI, body mass index; BP, blood pressure; DBP, diastolic blood pressure; eGFR, estimated glomerular filtration rate; Hb1Ac, hemoglobin A1c; LDL, low-density lipoprotein; SBP, s~~S~~ystolic blood pressure.
